# Emergence of supercontraction in regenerated silkworm (*Bombyx mori*) silk fibers

**DOI:** 10.1038/s41598-019-38712-6

**Published:** 2019-02-20

**Authors:** José Pérez-Rigueiro, Rodrigo Madurga, Alfonso M. Gañán-Calvo, Manuel Elices, Gustavo V. Guinea, Yugo Tasei, Akio Nishimura, Hironori Matsuda, Tetsuo Asakura

**Affiliations:** 1 Centro de Tecnología Biomédica, Universidad Politécnica de Madrid, 28223 Pozuelo de Alarcón, (Madrid), Spain; 2 Departamento de Ciencia de Materiales, ETSI Caminos, Canales y Puertos, Universidad Politécnica de Madrid, 28040 Madrid, Spain; 3 Biomedical Research Networking Center in Bioengineering, Biomaterials and Nanomedicine (CIBER-BBN), Madrid, Spain; 4 Escuela Técnica Superior de Ingenieros, Universidad de Sevilla, 41092 Sevilla, Spain; 5 Department of Biotechnology, Tokyo University of Agriculture and Technology, 2-24-16 Nakacho, Koganei, Tokyo 184-8588 Japan

## Abstract

The conditions required for the emergence of supercontraction in regenerated silkworm (*Bombyx mori*) silk fibers are assessed through an experimental approach that combines the spinning of regenerated fibers with controlled properties and their characterization by ^13^C solid-state nuclear magnetic resonance (NMR). Both supercontracting and non-supercontracting regenerated fibers are produced using the straining flow spinning (SFS) technique from ^13^C labeled cocoons. The short-range microstructure of the fibers is assessed through ^13^C CP/MAS in air and ^13^C DD/MAS in water, and the main microstructural features are identified and quantified. The mechanical properties of the regenerated fibers and their microstructures are compared with those of natural silkworm silk. The combined analysis highlights two possible key elements as responsible for the emergence of supercontraction: (1) the existence of an upper and a lower limit of the amorphous phase compatible with supercontraction, and (2) the existence of two ordered phases, β-sheet A and B, which correspond to different packing arrangements of the protein chains.

## Introduction

Silks^1^ are remarkable materials when considered in terms of their processing route, microstructure and properties. Silk fibers are produced by some groups of arthropods, most conspicuously spiders and moths (*Lepidoptera*), for such critical biological functions as protecting the offspring or building the web^2^. In spite of having appeared as the result of two independent evolutionary events^3^, silks from spiders and worms share an extensive number of features in terms of sequence^4,5^, spinning mechanism^6,7^, mechanical properties^8,9^ and biocompatibility^10^.

Probably the most salient feature in which silks from spiders and silkworms differ is the existence of the supercontraction phenomenon in the former. Supercontraction is a characteristic feature first identified in the major ampullate gland (MA) silk of Orbicularian spiders. Initially, the term supercontraction was used to refer to the significant reduction of its length when the fiber is immersed in water with at least one of the ends unrestrained^11^. It was later realized that the shrinkage of the fiber is simply the most evident effect of a much more profound property of the material: the existence of a ground state to which the fiber can revert independently from its loading history by immersion in water^12,13^. The existence of such a ground state implies that the fiber can be stretched following an arbitrary sequence of loads, but it will recover the tensile behaviour of this ground state by being simply allowed to contract in water. Later studies showed that spider silks different from MA, such as flagelliform silk, also exhibited a ground state^14^ and that supercontraction extended outside the Orbicularian group^15,16^.

In contrast to this wide distribution of the supercontraction phenomenon among spider silk fibers, silkworm (*Bombyx mori*) silk presents a negligible shrinkage when immersed in water which, in addition, is not related to the existence of a ground state in the material^17,18^. Consequently, the possibility of producing regenerated silkworm silk fibers with the composition of silkworm silk, but endowed with the property of supercontraction^19^ was considered as a nice opportunity to understand the principles that underlie the emergence of this phenomenon.

Prior to the production of supercontracting regenerated silkworm silk fibers, the basic strategy for analysing the origin of this phenomenon relied on the comparison of the sequences and microstructures of natural fibers, either endowed or not with this property^20–22^. Silks such as those produced by the major and minor ampullate glands of the spiders and that produced by silkworms (*B. mori*) were included in the analysis, but no clear correlation could be established to relate the microstructures of the different fibers and the emergence of supercontraction^23–25^. In particular, solid-state nuclear magnetic resonance (NMR) was a preferred technique for the microstructural characterization of silk fibers, since it allows unveiling some singular microstructural features in the short-range organization of the fibers^26–32^.

Following these ideas, this work exploits the versatility of the straining flow spinning (SFS) technique^33,34^ to (1) tune the appearance of supercontraction in regenerated silk fibers by modifying the processing parameters, and (2) produce the relatively large amount of material required for solid-state NMR analysis. Thus, supercontracting and non-supercontracting regenerated silkworm silk fibers were produced by simply applying (or not) a post-spinning drawing step in water to regenerated SFS fibers. The mechanical properties of the fibers were characterized both in air and immersed in water in the as spun and supercontracted states, and their short range organization was explored with the unique insight offered by ^13^C solid-state NMR^32^. Both the mechanical properties and NMR characterization were compared with the data obtained from the natural fibers^23^. As shown below, this combined experimental approach allows identifying the microstructural features that may underlie the emergence of supercontraction. The identification of these features might have far reaching consequences, since it would offer the possibility of incorporating this property to a whole range of new artificial fibers and lead to the development of novel applications.

## Results

### Tensile behaviour of natural (N) and SFS regenerated (R-) silkworm silk fibers

**Figure 1:**
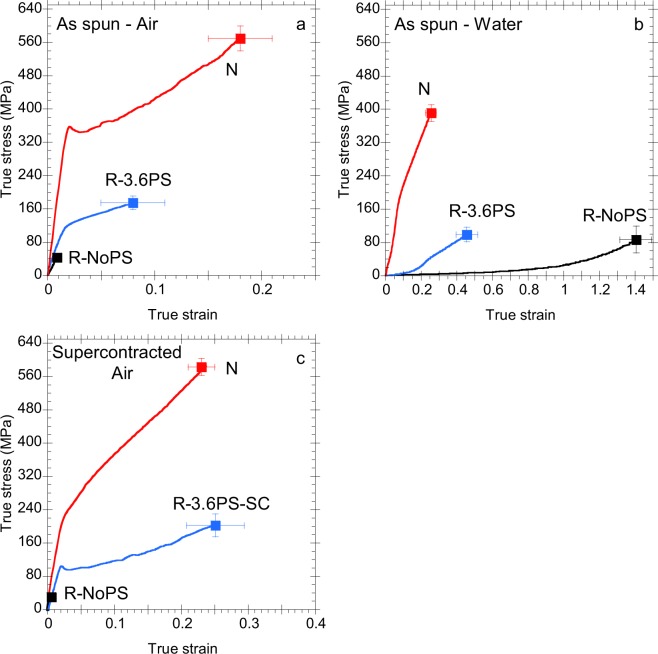
True stress-true strain curves of N (red), R-NoPS (black) and R-3.6PS (blue) fibers. (**a**) As spun fibers tested in air, (**b**) as spun fibers tested in water, and (**c**) fibers immersed in water, allowed to contract, dried and tested in air. A representative curve for each material and testing condition is presented, on which the average values of tensile strength and strain at breaking of at least three tests are indicated by a square symbol. Error bars correspond to the standard errors of the tensile strength and strain at breaking for each material and testing condition. Data of the N fibers in (**a**,**c**) are reproduced from the reference^9^. Data of the N in (**b**) are reproduced from the reference^35^.

In spite of sharing a similar composition, natural (N) and regenerated (R-) silkworm (*Bombyx mori*) silk fibers may exhibit very different tensile properties as illustrated in Fig. 1. The true stress-true strain curves of N, R-NoPS and R-3.6PS regenerated silk fibers tested in air are compared in Fig. 1a. It is apparent that N fibers outperforms their artificial counterparts in terms of elastic modulus and tensile strength as found in a number of previous studies^17,18^. It was found, however, that regenerated silk fibers may reach higher values of strain at breaking under adequate processing conditions^19^. These higher values of strain at breaking, in turn, lead to values of work to fracture in some regenerated fibers^19,33^ that are comparable to those of the natural material.

Differences between the tensile behaviour of natural and regenerated silks are even more apparent when the fibers are tested in water. Figure 1b compares the true stress-true strain curves of N, R-NoPS and R-3.6PS fibers tested in water. As illustrated in Fig. 1b, immersion in water of N fibers induces a moderate reduction in the elastic modulus and tensile strength, while the strain at breaking is not affected. The effect of water on N fibers is explained by the collapse of the hydrogen bonds established between the fibroins in the dry fiber. In the wet fiber the protein-protein hydrogen bonds initially present in the dry fiber are substituted by water-protein hydrogen bonds, which do not contribute to the tensile behaviour of the fiber. In this regard, the mechanical behaviour of the wet fiber is controlled by the stiff β-nanocrystals and by the protein chains that interact among them and with the β-nanocrystals through van der Waals forces^35^.

In comparison, water exerts more extreme changes in regenerated silkworm silks, although the details depend on the concrete processing parameters^23^. In this case, both R-NoPS and R-3.6PS samples tested in water show a significant reduction in the elastic modulus (over two orders of magnitude for R-NoPS samples), and an increase in the strain at breaking. Besides, the regenerated fibers tested in water show tensile properties characteristic of an elastomer as occurs with MA spider silk tested in water^36^. The effect of water on the regenerated silk fibers was explained as a result of the collapse of the hydrogen bond network found in the dry fiber, so that protein chains with an elastomeric behaviour control the mechanical behaviour of the wet material^36–38^. In this regard, the true stress-true strain curves of the regenerated fibers tested in water are more similar to those of major ampullate spider silk (MAS)^11^ than to the natural silkworm silk from which the regenerated fibers are produced.

The similarity between spider silk and regenerated silkworm silk fibers suggests exploring the possible existence of supercontraction in the latter. Figure 1c compares the true stress-true strain curves of the fibers after being immersed in water, allowed to contract and dried. No contraction is measured in the R-NoPS samples and their tensile properties concur with those of the as spun fibers. Natural silk shows a small contraction (~1–2%)^17^, but immersion in water and subsequent drying modifies the true stress-true strain curve. This modification concentrates around the yield point and results from changes in the hydrogen bond network, mainly in the amorphous regions of the material, and subsequent conformational changes of the fibroin proteins^9^. As observed in Fig. 1a,b, the most evident change in the tensile properties of N fibers upon immersion in water and subsequent drying is a significant reduction in the yield stress of the material.

**Figure 2:**
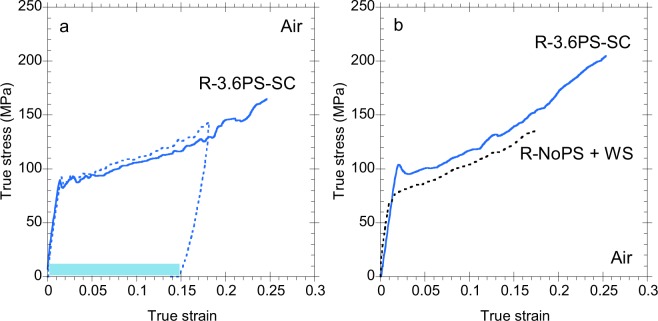
(**a**) A representative recovery test of a R-3.6PS-SC regenerated silk fiber. The maximum supercontraction step between the first and the second tensile tests is indicated by the light blue rectangle. (**b**) Comparison of the true stress-true strain of a R-3.6PS regenerated silk fiber after maximum supercontraction (R-3.6PS-SC) and an R-NoPS regenerated silk fiber subjected to wet stretching.

In contrast to both N and R-NoPS samples, R-3.6PS regenerated silk fibers show a contraction of 17% from the original length. In addition, a significant increase in the strain at breaking is observed after the R-3.6PS samples are allowed to contract, resulting in a work to fracture of W_f_ = 34 MJ/m^3^ for these samples. As indicated above, the contraction of a silk fiber in water is just an initial hint of the supercontraction ability of the material. In order to substantiate this property, it is necessary to prove the existence of a true ground state by using recovery tests^39^. Four recovery tests were performed on R-3.6PS-SC samples and a representative recovery test is shown in Fig. 2a. In an initial step, the fiber is stretched in air up to a strain of 0.18. Then, the fiber is unloaded and allowed to contract in water (this step is indicated in the Figure by the light blue rectangle) and, finally, tensile tested until breaking. The concurrence of the true stress-true strain curves of both tensile tests observed in Fig. 2a is considered as the definitive evidence of a ground state in R-3.6PS samples. From Fig. 2a it is also apparent that the properties of the as-spun R-3.6PS sample are recovered upon stretching the R-3.6PS-SC sample in air.

In spite of the differences in the true stress-true strain curves of the R-3.6PS and R-NoPS samples when tested in air, R-3.6PS are obtained from R-NoPS samples upon adding a post-spinning drawing step during processing. Thus, the similarity of the mechanical behaviour of R-3.6PS and R-NoPS samples when tested in water hints to the existence of some common underlying microstructural features between both types of fibers.

The existence of supercontraction in R-3.6PS fibers, which reflects the significant structural flexibility of the material, supports the possibility of finding a procedure to interconvert the microstructure and properties of both types of regenerated silks. In this regard, it was found that the tensile properties of regenerated silk fibers prepared from an N-methyl morpholine oxide (NMMO) solution can be modified through a wet stretching process^17^, in parallel with the behaviour of MA spider silk^40^. Following this rationale, R-NoPS samples were subjected to wet stretching and allowed to contract. The true stress-true strain curve of a wet-stretched R-NoPS (R-NoPS + WS) sample is compared with that of a R-3.6PS-SC sample in Fig. 2b. The concurrence of both true stress-true strain curves indicates that R-NoPS fibers are converted into R-3.6PS-SC fibers through a wet-stretching process.

The comparison of the distinct behaviour of the N, R-NoPS, R-3.6PS and R-3.6PS-SC silk fibers offers an intriguing scenario. Firstly, both natural and regenerated fibers share essentially a common composition, so that the observed differences must be related with their different processing routes and microstructures. Secondly, in spite of their diverging mechanical properties, R-NoPS, R-3.6PS and R-3.6PS-SC samples share a common composition and processing conditions, except for the absence of a post-spinning drawing step in water during the spinning of the R-NoPS samples. In addition, it is also found that the behaviour of the R-NoPS samples can be modified to concur with that of the R-3.6PS-SC fibers through a simple wet-stretching process. Finally, R-3.6PS and R-3.6PS-SC fibers can be interconverted into one another by stretching in air and maximum supercontraction, respectively.

In this context, the connections among these natural and regenerated fibers offers a unique opportunity to explore the relationships between microstructure and properties in silk fibers, and NMR arises as a powerful microstructural characterization technique especially adequate for this task. In particular, usage of ^13^C labelled samples allows (1) isolating the structural information obtained from different amino acids and, consequently, distinguishing between crystalline and amorphous regions, (2) obtaining microstructural information on the effects related with the interactions between silk proteins and water molecules, and (3) obtaining information on the dynamics of the different microstructural features.

### ^13^C CP/MAS NMR spectra of natural (N) and regenerated (R-) SF fibers in the dry state

**Figure 3:**
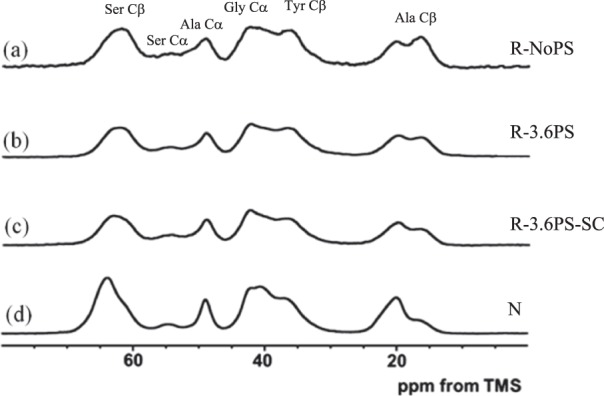
^13^C CP/MAS NMR spectra of (**a**) R-NoPS, (**b**) R-3.6PS, (**c**) R-3.6PS-SC and (**d**) N fibers in the dry state.

Figure 3 shows representative ^13^C CP/MAS NMR spectra of [3-^13^C] Ser-, [3-^13^C] Tyr- and [3-^13^C] Ala-labeled (a) R-NoPS, (b) R-3.6PS, (c) R-3.6PS-SC and (d) N fibers, in the dry state together with the assignment of the main peaks.

The selective ^13^C labeling of the Tyr Cβ and Ser Cβ carbons makes possible to study the local conformation of these residues in detail^41,42^. The assignment of Tyr Cβ, Ser Cβ and Ala Cβ peaks is shown in Fig. 3(a), although the naturally abundant Gly Cα peak overlaps at the lower field with the Tyr Cβ peak. Structural and dynamical information on the crystalline domain can be obtained from the Ser peak and that of the non-crystalline domain from the Tyr peak independently.

**Figure 4:**
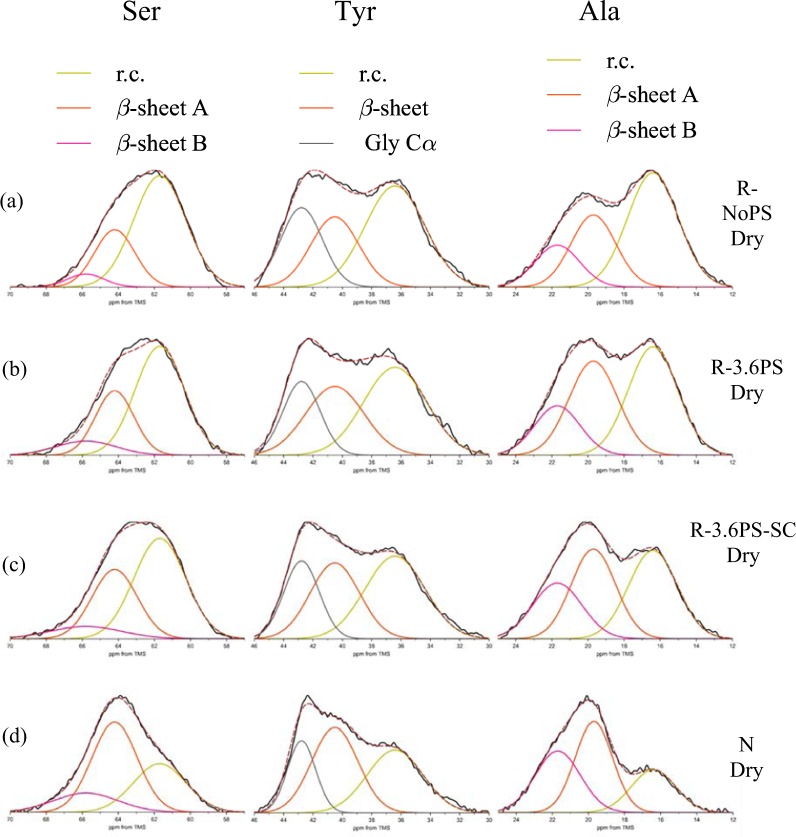
The expanded Cβ peaks in ^13^C CP/MAS spectra of Ser, Tyr and Ala residues of (**a**) R-NoPS, (**b**) R-3.6PS, (**c**) R-3.6PS-SC and (**d**) N fiber samples in dry state together with the deconvolution of the peaks.

Figure 4 shows the expanded Cβ peaks of Ser, Tyr and Ala residues in ^13^C CP/MAS NMR spectra of the R-NoPS, R-3.6PS, R-3.6PS-SC and N fibers, together with the deconvolution of the peaks.

**Figure 5:**
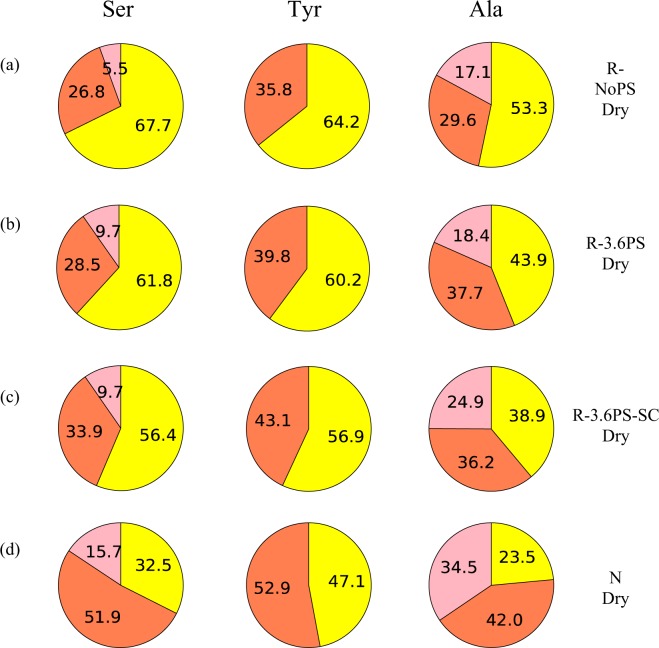
Pie charts of the fractions corresponding to the different conformations of Ser, Tyr and Ala Cβ peaks in the ^13^C CP/MAS NMR spectra of (**a**) R-NoPS, (**b**) R-3.6PS, (**c**) R-3.6PS-SC and (**d**) N fiber samples in the dry state obtained from the deconvolution of the NMR peaks. The color code is: yellow- random coil; red- β-sheet or β-sheet A; pink- β-sheet B.

Two main contributions can be identified in all the spectra, which correspond to β-sheet and random coil (r.c.) conformation. β-sheet, in turn, comprises two kinds of packing arrangements, which are labelled as β-sheet A and B. The interpretation of the latter β-sheet A and B is given in Fig. S1 (Supporting Information) and corresponds to differences in the packing arrangement of the β-sheet secondary structure. The deconvolution of the spectra to determine the fraction of the various conformations for each residue followed the method presented in previous papers^23,42^. In particular, the chemical shifts of the ^13^C NMR spectra were used to identify each elementary contribution^28–32,41^. The fractions of the different conformations obtained from the deconvolution are shown in Fig. 5, and the values are summarized in Table S1 (Supporting Information).

The fraction of β-sheet increases in the order of R-NoPS, R-3.6PS, R-3.6PS-SC and N fiber for Ser and Ala residues. The Tyr residue also shows a similar tendency, but changes are comparatively smaller.

### ^13^C DD/MAS NMR spectra of natural (N) and regenerated (R-) SF fibers in the hydrated state

**Figure 6:**
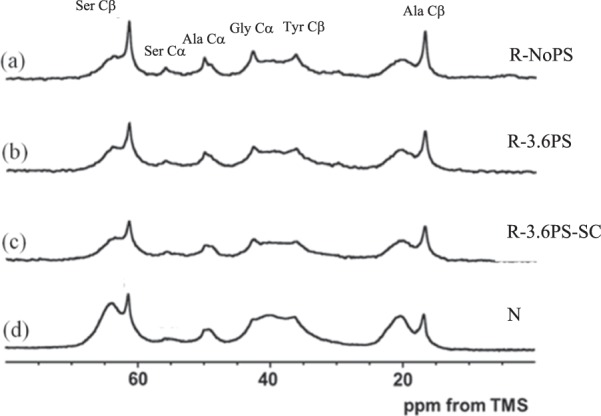
^13^C DD/MAS NMR spectra of (**a**) R-NoPS, (**b**) R-3.6PS, (**c**) R-3.6PS-SC and (**d**) N samples in the hydrated state.

Hydration leads to a more heterogenous structure that reflects the different effect of the presence of water molecules on the individual domains of the silk fibers. In order to analyze the spectra of the hydrated samples quantitatively, ^13^C DD/MAS NMR was used instead of ^13^C CP/MAS NMR, although there is no significant difference between ^13^C DD/MAS NMR and ^13^C CP/MAS NMR spectra of *B. mori* silk fibroin fiber in the dry state^42^. Figure 6 shows ^13^C DD/MAS NMR spectra of [3-^13^C] Ser-, [3-^13^C] Tyr- and [3-^13^C] Ala-labeled (a) R-NoPS, (b) R-3.6PS, (c) R-3.6PS-SC and (d) N fibers in the hydrated state.

**Figure 7:**
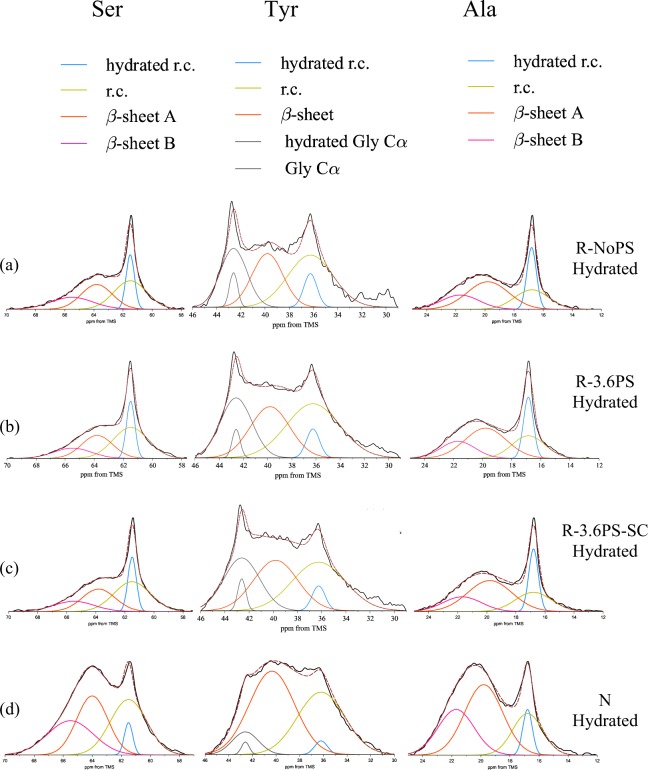
The expanded Cβ peaks of Ser, Tyr and Ala residues of (**a**) R-NoPS, (**b**) R-3.6PS, (**c**) R-3.6PS-SC and (**d**) N fiber samples in the hydrated state together with the deconvolution of the peaks. In addition to the contributions previously identified from the ^13^C CP/MAS NMR spectra, a new hydrated random coil contribution is identified in the wet samples using ^13^C DD/MAS NMR.

A remarkable difference between the spectra of the dry and hydrated SF samples is the appearance of sharp peaks in the spectra of the latter. These peaks can be assigned to the hydrated random coil peaks^23,42^, and result from the increased mobility of some protein regions upon interaction with water molecules. The appearance of the sharp peaks was observed for all residues  characterized in Fig. 6. Detailed analyses were performed from the deconvoluted spectra as shown in Fig. 7.

**Figure 8:**
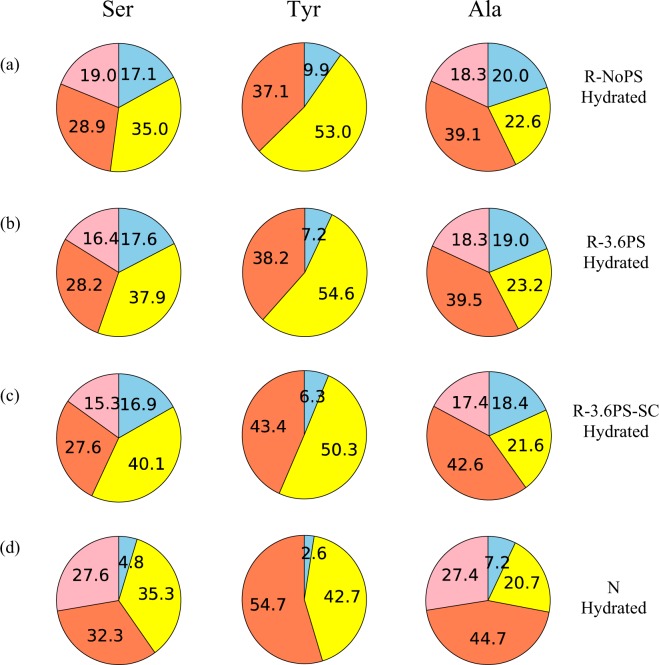
Pie charts of the fractions corresponding to the different conformations of Ser, Tyr and Ala Cβ peaks in the ^13^C DD/MAS NMR spectra of (**a**) R-NoPS, (**b**) R-3.6PS, (**c**) R-3.6PS-SC and (**d**) N fiber samples in the hydrated state obtained from the deconvolution of the NMR peaks. The color code is: yellow-random coil; light blue- hydrated random coil; red- β-sheet or β-sheet A; pink- β-sheet B.

The fractions of the various conformations obtained from the deconvolution are shown in Fig. 8, and their values are summarized in Table S2 (Supporting Information).

Relatively small differences are found between the fractions of the four components in the regenerated silk fibers. In contrast, significant differences are observed when the regenerated fibers are compared with N samples. In particular, N fibers show a smaller proportion of hydrated random coil, and a higher proportion of β-sheet B.

## Discussion

As indicated previously, the characterization of the system formed by R-NoPS, R-3.6PS, R-3.6PS-SC and N fibers offers a privileged opportunity for finding correlations between the singular properties of these materials and some of their microstructural features. This opportunity is especially clear with regard to the emergence of supercontraction in silk fibers, due to the possibility of spinning both supercontracting and non-supercontracting regenerated fibers with the composition of (non-supercontracting) natural silkworm silk by simply switching on and off a post-spinning drawing step.

Application of NMR to the study of this set of fibers allows exploring the relationship between their short-range microstructural features and the existence (or not) of supercontraction^23,31,32^. It must be acknowledged that some long-range microstructural features, such as the size or orientation of the β-nanocrystals, might also play a role in the appearance of supercontraction. Long-range microstructural details are outside the scope of NMR, but previous works on the analysis of these feature did not allow reaching any firm conclusion on its influence on the origin of this phenomenon^19,43^.

The study of the NMR spectra of N fibers represents a convenient starting point for the analysis of the whole set of natural and regenerated fibers, since a number of previous works^23,32,44–47^ have established the main microstructural features of this material. In addition, such a study takes advantage from the possibility of characterizing the fibers both dry and wet.

Silkworm silk presents a semicrystalline microstructure in which β-nanocrystals are embedded in an amorphous matrix. In broad terms it is assumed that β-nanocrystals impart silkworm silk its structural integrity and stiffness, while amorphous regions are responsible for its remarkable strain at breaking. The singular sequence of silkworm silk^4^ is critical for the appearance of the crystals from regions with β-sheet secondary structure, since they are formed from the characteristic motif of sequence –GAGAGS–. The relationship between this motif of sequence and the crystals implies that Ser residues appear mostly in the crystalline phase, Tyr residues in the amorphous phase, and Ala residues can appear in both phases. Immersion in water of silkworm silk fibers leads to a very small contraction, which is not associated with the existence of a ground state (i.e. N fibers does not show proper supercontraction). It was found that the crystallinity of silkworm silk fibers increases when first immersed in water^9^, but subsequent wetting/drying cycles do not modify either the structure further or lead to an increase in the number of the crystals^48^.

As expected from the large proportion of –GAGAGS– motifs in its sequence, silkworm silk shows one of the highest crystallinities (~60%) of all silks^48^. This high crystallinity is consistent with the β-sheet contributions to the Ala- and Ser-NMR peaks (Figs 4 and 7), although the interpretation of these data should take into account that NMR provides information on the secondary structure of the amino acids, but does not distinguish between amino acids present in the amorphous or in the crystalline phase. It is also found that β-sheet A constitutes the major contribution to this secondary structure, which is consistent with its participation in the formation of β-nanocrystals. The absence of significant macroscopic changes in N upon immersion in water is best reflected by the constancy of the random coil and β-sheet contributions in the Tyr-NMR peaks. In contrast, a tendency towards increasing the random coil structure at the expense of the β-sheet structures is observed in the Ala- and Ser-peaks upon wetting.

Significant differences are found when the NMR spectra of the R-NoPS samples are compared with those of N. In R-NoPS samples the random coil structure represents more than 50% of the total area of all peaks in the dry state. Upon wetting a significant fraction of the random coil structure is converted into hydrated random coil and into β-sheet.

The comparison of the NMR data from the N and R-NoPS fibers suggests that a minimum proportion of random coil structure is required to elicit the elastomeric behaviour of the fibers when tested in water. Additionally, it might also be critical that a sufficient fraction of the random coil becomes hydrated when the material is immersed in water. The requirement of a minimum proportion of random coil to impart the fiber the ability to supercontract is consistent with previous results on regenerated fibers^23^, in which spinning proceeded from a solution of silk protein in hexafluoroisopropanol (HFIP) and methanol (MeOH) was used as coagulant. The ^13^C CP/MAS NMR spectra of [3-^13^C] Ser-, [3-^13^C] Tyr- and [3-^13^C] Ala-labeled of these regenerated fibers found a large proportion of β-sheet, comparable to that in N samples, even in the case of as spun (no-stretched) fibers. No supercontraction was found upon immersion of these fibers in water.

The conversion of random coil into β-sheets in the wet fibers suggests that the driving force behind the observed microstructural changes in the R-NoPS fibers might be the tendency to increase crystallinity at the expense of the amorphous phase. A comparable tendency to increase the values of crystallinity upon wetting and drying is found in N and explains the effects that degumming exerts on silkworm silk^9^.

When the previous discussion is extended to include the R-3.6PS and R-3.6PS-SC, some regularities are immediately found. In the dry fibers the proportion of β-sheet follows the order: N > R-3.6PS-SC > R-3.6PS > R-NoPS for all three NMR peaks (correspondingly, the proportion of random coil follows the order R-NoPS > R-3.6PS > R-3.6PS-SC > N). When the wet fibers are considered it is found that the proportion of hydrated random coil follows the order: R-NoPS ~ R-3.6PS ~ R-3.6PS-SC > N. Consistently with the previous hypothesis on the origin of the elastomeric behaviour in regenerated fibers upon immersion in water, R-3.6PS and R-3.6PS-SC fibers show a larger proportion of random coil structure compared with N fibers, and the fraction of hydrated random coil is comparable to that of the R-NoPS sample.

At this point of the discussion, the emergence of supercontraction in the R-3.6PS samples is probably the most intriguing question of the system formed by the four sets of fibers. The comparison of the natural and regenerated fibers supports the hypothesis that high values of crystallinity may limit the conformational changes induced on the fiber by wetting. In turn, these restrained conformational changes would prevent the N fibers from supercontrating. Consequently, there seems to exist a maximum threshold to the crystallinity of the fibers that allows the appearance of supercontraction. In addition, and although tempting, the comparison of the R-3.6PS samples with the R-NoPS fibers immediately precludes establishing a simple relationship between the proportion of random coil structure and supercontraction. In this context, such a direct relationship would imply that R-NoPS samples should not only supercontract, but also that their percentages of supercontraction should be possibly higher than those of the R-3.6PS samples. Thus, there seems to exist a “sweet spot” in the microstructural organization that allows the emergence of supercontraction. The previous data suggest that the “sweet spot” would be defined by the existence of a lower and an upper limit to the proportion of the amorphous phase. The existence of a lower limit is compatible with the data obtained from the N material, while that of the upper limit is indicated by the results on R-NoPS fibers.

Fortunately, the possibility of converting R-NoPS into R-3.6PS-SC fibers through wet-stretching and of interconverting R-3.6PS and R-3.6PS-SC fibers through supercontraction/stretching in air offers the possibility of getting a deeper insight on the conditions required by supercontraction. The NMR data of the dry fibers indicate that the transition from R-NoPS to R-3.6PS-SC samples implies the conversion of random coil structures to β-sheets both in the A and B forms. In addition, the comparison of the R-3.6PS-SC and R-3.6PS spectra indicates that the stretching of the R-3.6PS-SC leads to an increase in the proportion of random coil at the expense of the β-sheet structure. The higher proportion of β-sheet structure after supercontraction found in the R-3.6PS-SC compared to R-3.6PS samples is again compatible with the consideration of the increase in the crystallinity of the material as the major driving force that causes the observed microstructural changes.

Another major difference is also found between the R-NoPS and the R-3.6PS-SC samples when the effect of water is considered. As shown in Figs 5 and 8 and in Tables S1 and S2 (Supplementary Information) and, in contrast to the conversion of random coil to β-sheet observed from the dry to the wet states of R-NoPS fibers, R-3.6PS-SC only show the interconversion between the β-sheet A and B forms, but no net conversion between random coil and β-sheet structures.

Following the previous discussion, the development of a comprehensive scheme that encompasses the behaviour and properties of both natural and regenerated fibers requires providing an explanation for the absence of supercontraction in R-NoPS samples. A possible hypothesis for this behaviour can be found in the principles of the elastomeric behaviour of polymer chains. The one-dimensional constitutive equation of an elastomeric material^37,38^ is:

1 $${\rm{\sigma }}=1/3{{\rm{EN}}}^{1/2}{ {\mathcal L} }^{-1}({\rm{\lambda }}/{{\rm{N}}}^{1/2})$$

where E is the elastic modulus of the material (E = nk_B_T, with n the density of chains, defined as the number of polymer chains per unit volume), N is the number of links of each chain and the elongation, λ, is defined from the initial length, L_0_, and the instantaneous length, L, as: λ = L/L_0_, k_B_ is Boltzmann’s constant and ${\mathcal L}$ ^−1^ is the inverse of Langevin function. This constitutive equation implies that the elastomeric behaviour is favoured by increasing the density of polymer chains and the number of links of each chain. Consistently with this behaviour, the elastomeric behaviour of regenerated fibers in water is associated with a larger proportion of random coil structure which, in addition, can be hydrated. This model is consistent with the lack of supercontraction in the highly crystalline natural silkworm silk, and suggests the existence of a lower limit in terms of the density of chains and of the length of the chains compatible with the appearance of an elastomeric behaviour in water and with the phenomenon of supercontraction.

In addition to the existence of these lower limits, the distinct behaviour of the R-NoPS and R-3.6PS regenerated samples is consistent with the existence of upper limits to these parameters. In this case the limit might be imposed by the conformational freedom that the protein chains require to refold, which is the basis of the elastomeric behaviour and of supercontraction in spider silk fibers^49^. In effect, an increase in the density of chains and/or in the length of the chains would increase the possibility of creating entanglements between the chains^37,38^, whose presence would limit the conformational freedom of the protein chains and would prevent the material from exhibiting the properties of an elastomer. In this context, the existence of two ordered phases, β-sheet A and B, which correspond to different packing arrangements of β-sheets might also play a role in the appearance of supercontraction.

A particularly deep insight arises when the previous discussion is extended to include the supercontraction effect in natural fibers first described in the major ampullate gland silk of orbicularian spiders^11^. Firstly, the comparison of the sequences of the major ampullate gland and silkworm silks reveals a number of parallelisms and differences. Both fibers are composed of large proteins (in excess of 300 kDa) formed by the repetition of a few motifs of sequence. As indicated above, the basic motif of sequence of silkworm silk is –GAGAGS–. In contrast, and although the motif –GA– appears in some spider silk proteins (spidroins), the main motifs of the latter are –A_n_–, –GGX– (where X stands for an amino acid of a limited set that includes glutamine and tyrosine), and –GPG–^5^. The accepted assignment of the role of each motif in the microstructural features and behaviour of spider silk was the result of a detailed analysis of silks with different proportions of these motifs. Thus, it was found that the motif –A_n_– is responsible for the formation of the β-nanocrystals in spider silk^50^ (i.e. a similar role to that of the –GAGAGS– motif in the β-nanocrystals of silkworm silk). In contrast, the –GGX– motif is thought to be responsible for the appearance of supercontraction in spider silk fibers^51^, although supercontraction is potentiated by the presence of the proline-containing motif –GPG–^52,53^.

Although a comprehensive understading of supercontraction in spider silk is missing, some aspects of the dynamics of the process were identified from microstructural analyses on supercontracted fibers, mostly involving XRD and NMR characterization. Thus, β-nanocrystals were shown to rotate with respect to the axis of the fiber as a result of supercontraction, but their size remained basically stable upon immersion in water^54^ as identified by XRD. The subsequent discovery of two distinct arrangements in the β-nanocrystals^55^, labelled as “rectangular” and “staggered”, suggests that the existence of two slightly different ordered phases in the fiber might favour the appearance of supercontraction in major ampullate gland silk. The main effect of immersion in water on the non-β-crystalline regions was found to be the collapse of the hydrogen bond network that appears in the dry fiber, so that the elastomeric properties of protein chains are exhibited^37,38^. It was observed that stretching the fiber in water leads to an increase in the crystalline fraction, in spite of the dimensional stability of the β-nanocrystals. Such a behaviour is also observed in flagelliform silk^14^, where it was found to be the result of the formation of polyproline II nanocrystals. The similarities in the sequence of major ampullate gland and flagelliform silks suggests that a similar mechanism might occur in the former, although a direct experimental evidence of the formation of polyproline II nanocrystals in major ampullate gland silk is lacking.

The comparison of the microstructure and dynamics of natural spider silk and regenerated silkworm silk fibers allows establishing some common principles, but also some differences in the supercontraction of the two types of fibers. In this regard, there seems to exist a maximum limit to the crystalline fraction in both types of fibers, although this limit is different for natural spider and regenerated silk fibers. In natural spider silk it was found that fibers with a crystallinity in excess of 20% did not supercontract^22,56^. In addition, an inverse correlation was proposed between crystallinity and the percentage of supecontraction in this material. The data presented in this work do not yield a direct determination of the crystalline fraction, but previous studies have established a value of crystallinity of ~60% for natural silkworm silk^48^, while regenerated silkworm silk fibers with a crystallinity of ~20%^19^ exhibited supercontraction. These results suggest that the maximum threshold of crystallinity compatible with the appearance of supercontraction depends on the sequence, and consequently on the detailed elastomeric properties of the protein chains. However, and as also found in regenerated silk fibers, a low crystallinity does not necessarily result in supercontracting fibers. For instance, the silk of the Mygalomorph spider *Aphonopelma seemani* does not supercontract, in spite of a  crystallinity comparable to that of *Argiope aurantia*, whose major ampullate gland fibers show one of the largest percentages of supercontraction of the orb-weaving spiders^16^. In this case, the absence of supercontraction in the silk of *A. seemani* is supposed to be related with the differences in the sequence of the spidroins and, in particular, with the absence of the –GGX– and –GPG– motifs^8^.

In contrast, the data on regenerated silk fibers show that fibers with the same sequence may or may not exhibit supercontraction depending on the microstructural organization of the chains. As explained above, the absence of supercontraction in the R-NoPS is supposed to be related with the existence of a large number of entanglement between chains, if the percentage of the crystalline phase is below a lower limit. A similar negative effect on supercontraction was reported from spider silk fibers in which crosslinks between spidroins were formed by exposure to UV radiation^49^. The constraint imposed on the conformational freedom of the chains was found to be associated with a significant decrease in the percentage of supercontraction and in the strain at breaking of the fibers. Finally, the existence of two alternative ordered arrangements in the β-nanocrystals both in regenerated silkworm silk and in natural spider silk might also play a role in allowing a more ordered folding of the proteins, so that the possibility of creating entanglements during supercontraction were reduced.

## Conclusions

The previous analysis of the system formed by N, R-NoPS, R-3.6 PS and R-3.6PS-SC silk fibers offers a deep view of the relationship between the short-range microstructural details as explored by NMR and the mechanical properties of these materials. The results obtained are relevant for establishing correlations between these microstructural details and the emergence of supercontraction.

The NMR data show that all fibers share a common set of secondary structures: random coil, hydrated random coil (in the wet samples), β-sheet A and β-sheet B. Consequently, it can be concluded that differences in their mechanical properties, including the phenomenon of supercontraction, must depend on quantitative differences in the proportion of these contributions.

The existence of an upper limit to the proportion of ordered β-sheet phase in the material compatible with supercontraction is established from the comparison of the N and regenerated fibers. Below this limit, fibers exhibit an elastomeric behaviour when tested in water and, under given processing conditions, supercontraction. Although it would be tempting to establish a direct relationship between the proportion of random coil phase and supercontraction, the comparison of the R-NoPS and R-3.6PS samples indicate that there must be also an upper limit to the random coil phase of the material compatible with the appearance of this phenomenon. If the proportion of random coil phase exceeds this upper limit, the required conformational freedom that allows the unfolding or refolding of the chains would be compromised and the fiber would not show supercontraction. The existence of two slightly different ordered phases, β-sheet A and β-sheet B may further promote the ordered conformational changes of the proteins required for supercontraction.

All previous considerations are compatible with the existence of a *sweet spot* in the short-range microstructural organization of silks, which corresponds to a compromise between the proportion of random and ordered structures in the materials and allows the emergence of supercontraction. The application of these principles opens the possibility of designing new artificial fibers, even with compositions different from those of silks, that might be endowed with the ability to supercontract.

## Materials and Methods

### Preparation of ^13^C labelled *B. mori* silk fibroin

*B. mori* larvae were reared in the laboratory of the Tokyo University of Agriculture and Technology. The ^13^C labeling of SF was achieved biosynthetically by oral administration of an artificial diet with ^13^C-enriched amino acids to larvae of the fifth instar, as reported previously^41,57^. Briefly, the supplementary Tyr and Ser was mixed with 2.0 g of an artificial diet per day. The amount of ^13^C-labelled Tyr and Ser was 10 mg each on the fourth and fifth day of the fifth larval stage. To prevent transfer of Ser into Gly, 20 mg non-labeled Gly was also mixed with the artificial diet per day. Thus, the total amount of Tyr and Ser was 20 mg per silkworm. The ^13^C labeling of Ala Cβ carbon was observed due to transamination from [3-^13^C] Ser in silkworm. The ^13^C-labeled amino acids, [3-^13^C]Tyr, and [3-^13^C]Ser (each 99% enrichment), used for labeling of silk fibroin, were purchased from Cambridge Isotope Laboratories, Inc., Andover, MA USA.

### Preparation of the dope

Deionized water was used for the degumming of the ^13^C labelled cocoons in a ratio 1/50 (w/v). Degumming proceeded in an autoclave at 121 °C during 50 minutes. After degumming, silk was dissolved in an 8 M LiBr and 0.1 M NH_4_HCO_3_ solution for 4 hours at 37 °C to a concentration of 10% (w/v). LiBr was removed through dialysis using deionized water with Snake-skin dialysis tubes of 3.5 kDa molecular cutoff. Water was changed every 8 hours, and the whole dialysis process took 48 hours. The fibroin solution was centrifuged to remove any debris at 5000 rpm and 4 °C for 20 minutes. The final concentration of the dope (16% w/v) was reached through a reverse dialysis process for 18 hours at 4 °C using a PEG 8000 Da solution at a concentration of 15% in an aqueous 1 M CaCl_2_ solution.

### Spinning parameters

The geometrical and hydrodynamic parameters of the straining flow spinning process^58^ were fixed to: diameter of the orifice of the nozzle, D_1_ = 400 μm; diameter of the capillary, d_1_ = 150 μm; tapering angle at the end of the capillary, α = 90°; distance between the take-up and the post-spinning drawing rollers, L_R_ = 60 cm; flow rate of the dope, Q_d_ = 5 μl/min; flow rate of the focusing fluid, Q_f_ = 0.4 ml/min, and velocity of the take up roller, V_R1_ = 3.5 m/min. The coagulating bath and focusing fluid consisted of a solution of ethanol and 1 M acetic acid in water in a ratio 80:20. Fibers produced under these conditions without a post-spinning drawing step were labelled as **R-NoPS** samples. Alternatively, some processes included a post-spinning drawing step in water. The post-spinning drawing step is described through the draw ratio, DR, defined as DR = V_R2_/V_R1_, where V_R2_ is the speed of the post-spinning roller and V_R1_ is the speed of the take-up roller. A draw ratio of DR = 3.6 was used in all the spinning processes that included a post-spinning drawing step, and samples are labelled as **R-3.6PS**.

### Supercontraction of the fibers

The ability of the regenerated fibers to supercontract was first assessed by measuring the shrinkage of the fiber while immersed in water. The length of the fiber after being immersed in water, allowed to contract and dried, L_MS_, was used to calculate the percentage of supercontraction %SC as^12^:

2 $$ \% SC=\frac{{L}_{0}-{L}_{MS}}{{L}_{0}}\times 100$$

where L_0_ is the initial length of the fiber.

The existence of a ground state, which constitutes the defining mark of supercontraction, was determined through recovery tests^39^. A recovery test consists of stretching the fiber in air, allowing it to supercontract, stretching the fiber again in air and comparing the stress-strain curves obtained from both stretching steps. Concurrence of the stress-strain curves indicates that the fiber has the ability to supercontract. R-3.6PS samples subjected to supercontraction were labeled as **R-3.6PS-SC**.

### Wet-stretching process

Fibers subjected to a wet-stretching process^40^ were first immersed in water and allowed to contract. Subsequently, the fiber was stretched while still immersed in water and their ends fixed to a pre-determined length. Finally, the fiber was removed from water and allowed to dry overnight. Wet-stretching was applied to some R-NoPS fibers (i.e. fibers not subjected to a post-spinning drawing step during the spinning process), that were stretched up to a value of 92% of their strain at breaking in water.

### Mechanical characterization

At least three samples for each condition were tested using an Instron 4411 tensile testing machine. The details of the mechanical tests can be found elsewhere^59^. Briefly, fibers were mounted on aluminium foil frames (base length 10 mm) and the diameters of the regenerated fibers were measured using an optical microscope (Leica DMI 3000B). The force exerted on the fibers during testing was measured using a balance (Precisa XT220A, resolution 1 μN). Tensile tests proceeded at a constant speed of 1 mm/min either in air (nominal environmental conditions: T = 25 °C and RH = 35%) or immersed in water at 20 °C. Stresses were calculated assuming a circular cross-sectional area.

True stress, σ, was calculated from the measured force assuming that the volume of the silk fiber does not vary during the test^60^ with the expression:

3 $$\sigma =\frac{F}{A}=F\frac{L}{{A}_{0}{L}_{0}}$$

where A_0_ and L_0_ are the initial area and length of the sample, and A and L the instantaneous magnitudes.

True strain, ε, was calculated correspondingly as:

4 $$\varepsilon =Ln\frac{L}{{L}_{0}}$$

### ^13^C solid-state NMR observation

The ^13^C cross polarization/magic angle spinning (CP/MAS) NMR spectra of SF in the dry and hydrated states were recorded using a Bruker Avance 400 NMR spectrometer with a 4-mm double resonance MAS probe and a MAS frequency of 8.5 kHz at room temperature. The SF samples were carefully inserted into a zirconia rotor and sealed with a polytetrafluoroethylene (PTFE) insert to prevent dehydration of the hydrated samples during NMR measurement^23^. Typical experimental parameters for the ^13^C CP/MAS NMR experiments were 3.5 μs ^1^H 90° pulse, 1 ms ramped CP pulse with 71.4 kHz rf field strength, two pulse phase modulation (TPPM) ^1^H decoupling during acquisition, 2176 data points, 8 k scans, and 4 s recycle delay. Details of the NMR conditions for the ^13^C dipolar dephasing/magic angle spinning (DD/MAS) NMR experiments were described in our previous paper^42^ and comprised a recycle delay of 5 s and a ^13^C 90° pulse of 3.5 μs. Lorentzian line broadening of 20 Hz was used for both the ^13^C CP/MAS and DD/MAS NMR spectra. The ^13^C chemical shifts were calibrated externally through the methylene peak of adamantane observed at 28.8 ppm with respect to external tetramethylsilane (TMS) at 0 ppm. The peak deconvolution of the ^13^C CP/MAS NMR spectra of SF samples observed in the dry state and ^13^C DD/MAS NMR spectra observed in the hydrated state was performed to determine the fraction of several conformations^23,42^. The fractions were summarized together with the chemical shifts (δ) and full width at half maximum (FWHM) obtained from the peak deconvolution.

## Supplementary information

Emergence of supercontraction in regenerated silkworm (Bombyx mori) silk fibers

## Data Availability

The mechanical data can be requested to jose.perez@ctb.upm.es. The NMR data can be requested to asakura@cc.tuat.ac.jp.
