# Supplementary material for: Emergence of supercontraction in regenerated silkworm (Bombyx mori) silk fibers
Source: Sci Rep. 2019 Feb 20;9:2398. doi: 10.1038/s41598-019-38712-6 (PMC6382804; doi:10.1038/s41598-019-38712-6)
Supplement: Supplementary file 1 — Emergence of supercontraction in regenerated silkworm (Bombyx mori) silk fibers [file 41598_2019_38712_MOESM1_ESM.docx]

**Emergence of supercontraction in regenerated silkworm (*Bombyx mori*) silk fibers**

José Pérez-Rigueiro^1,2,3,*^, Rodrigo Madurga^1,2^, Alfonso M. Gañán-Calvo^4^, Manuel Elices^1,2^, Gustavo V. Guinea^1,2,3^, Yugo Tasei^5^, Akio Nishimura^5^, Hironori Matsuda^5^ and Tetsuo Asakura^5,*^

1. Centro de Tecnología Biomédica. Universidad Politécnica de Madrid. 28223 Pozuelo de Alarcón (Madrid). Spain.
2. Departamento de Ciencia de Materiales. ETSI Caminos, Canales y Puertos. Universidad Politécnica de Madrid. 28040 Madrid (Spain).
3. Biomedical Research Networking Center in Bioengineering, Biomaterials and Nanomedicine (CIBER-BBN). Madrid. Spain.
4. Escuela Técnica Superior de Ingenieros. Universidad de Sevilla. 41092 Sevilla. Spain.
5. Department of Biotechnology. Tokyo University of Agriculture and Technology. 2-24-16 Nakacho, Koganei, Tokyo 184-8588. Japan.

*Corresponding Authors:

[jose.perez@ctb.upm.es](mailto:jose.perez@ctb.upm.es)

asakura@cc.tuat.ac.jp

Supporting Information


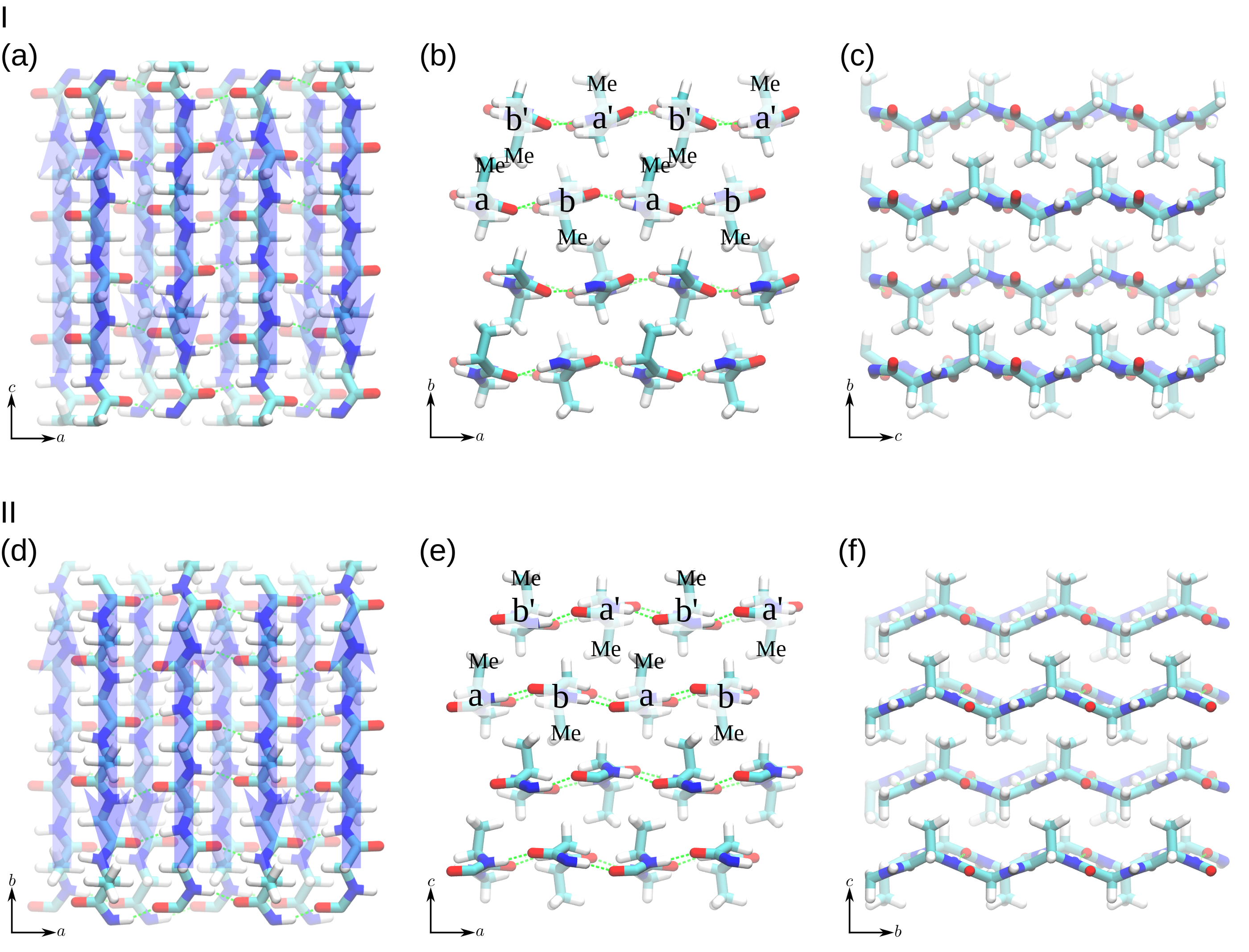


Figure S1. The β-sheet model I (top row) and model II (bottom row) for the crystalline regions of SF fiber with Silk II form, shown from three orthogonal orientations. The β -sheet A and B in Ala Cβ peaks in Figures 4, 5, 7 and 8 are assigned to the models I and II, respectively. The arrows in (a) and (d) represent (AG)_n_ sequences with the direction from the C-terminus to N-terminus. In the model I, the molecular axis is along the crystallographic axis c, and in the model II, the molecular axis is along the crystallographic axis b. The models I and II have different alternative antipolar arrangements for the four antiparallel β strands, a, b, and a’, b’, taking different directions of methyl groups (indicated by Me), as shown in (b) and (e)^29, 30, 51^.

Table S1

The fractions of the different conformations obtained from the deconvolution of ^13^C CP/MAS NMR spectra (Figure 5) of SF fibers in dry state.

| Ala |  | Random | β-sheet A | β-sheet B |
| --- | --- | --- | --- | --- |
|  | ppm | 16.8 | 19.6 | 21.7 |
|  | R-NoPS | 0.533 | 0.296 | 0.171 |
|  | R-3.6PS | 0.439 | 0.377 | 0.184 |
|  | R-3.6PS-SC | 0.389 | 0.362 | 0.249 |
|  | N | 0.235 | 0.420 | 0.345 |

| Tyr |  | Random | β-sheet |
| --- | --- | --- | --- |
|  | ppm | 36.1 | 40.3 |
|  | R-NoPS | 0.642 | 0.358 |
|  | R-3.6PS | 0.602 | 0.398 |
|  | R-3.6PS-SC | 0.569 | 0.431 |
|  | N | 0.471 | 0.529 |

| Ser |  | Random | β-sheet A | β-sheet B |
| --- | --- | --- | --- | --- |
|  | ppm | 61.5 | 64.0 | 65.5 |
|  | R-NoPS | 0.677 | 0.268 | 0.055 |
|  | R-3.6PS | 0.618 | 0.285 | 0.097 |
|  | R-3.6PS-SC | 0.564 | 0.339 | 0.097 |
|  | N | 0.325 | 0.519 | 0.157 |

Table S2

The fractions of the various conformations obtained from the deconvolution of ^13^C DD/MAS NMR spectra (Figure 8) of SF fibers in hydrated state.

| Ala |  | Random | Hydrated Ran | β-sheet A | β-sheet B |
| --- | --- | --- | --- | --- | --- |
|  | ppm | 16.8 | 16.8 | 19.6 | 21.7 |
|  | R-NoPS | 0.200 | 0.226 | 0.391 | 0.183 |
|  | R-3.6PS | 0.190 | 0.232 | 0.395 | 0.183 |
|  | R-3.6PS-SC | 0.184 | 0.216 | 0.426 | 0.174 |
|  | N | 0.207 | 0.072 | 0.447 | 0.274 |

| Tyr |  | Random | Hydrated Random | β-sheet |
| --- | --- | --- | --- | --- |
|  | ppm | 36.1 | 36.1 | 40.3 |
|  | R-NoPS | 0.530 | 0.099 | 0.371 |
|  | R-3.6PS | 0.546 | 0.072 | 0.382 |
|  | R-3.6PS-SC | 0.503 | 0.063 | 0.434 |
|  | N | 0.427 | 0.026 | 0.547 |

| Ser |  | Random | Hydrated Random | β-sheet A | β-sheet B |
| --- | --- | --- | --- | --- | --- |
|  | ppm | 61.5 | 61.5 | 64.0 | 65.5 |
|  | R-NoPS | 0.350 | 0.171 | 0.289 | 0.190 |
|  | R-3.6PS | 0.379 | 0.176 | 0.282 | 0.164 |
|  | R-3.6PS-SC | 0.401 | 0.169 | 0.276 | 0.153 |
|  | N | 0.353 | 0.048 | 0.323 | 0.276 |
